# Serum Erythritol and Risk of Overall and Cause-Specific Mortality in a Cohort of Men

**DOI:** 10.3390/nu16183099

**Published:** 2024-09-14

**Authors:** Jungeun Lim, Hyokyoung G. Hong, Jiaqi Huang, Rachael Stolzenberg-Solomon, Alison M. Mondul, Stephanie J. Weinstein, Demetrius Albanes

**Affiliations:** 1 Division of Cancer Epidemiology and Genetics, National Cancer Institute, National Institutes of Health, Bethesda, MD 20892, USA; jungeun.lim@nih.gov (J.L.); grace.hong@nih.gov (H.G.H.); jiaqi.huang@live.com (J.H.); rachael.solomon@nih.gov (R.S.-S.); weinstes@mail.nih.gov (S.J.W.); 2 Epidemiology and Community Health Branch, National Heart Lung and Blood Institute, National Institutes of Health, Bethesda, MD 20892, USA; 3 National Clinical Research Center for Metabolic Diseases, Key Laboratory of Diabetes Immunology, Ministry of Education, and Department of Metabolism and Endocrinology, The Second Xiangya Hospital of Central South University, Changsha 410011, China; 4 Department of Epidemiology, University of Michigan School of Public Health, Ann Arbor, MI 48109, USA; amondul@umich.edu; 5 Rogel Cancer Center, University of Michigan, Ann Arbor, MI 48109, USA

**Keywords:** erythritol, mortality risk, cardiovascular disease, heart disease, stroke, cancer

## Abstract

Erythritol occurs naturally in some fruits and fermented foods, and has also been used as an artificial sweetener since the 1990s. Although there have been questions and some studies regarding its potential adverse health effects, the association between serum erythritol and long-term mortality has not been evaluated. To examine the association between serum erythritol’s biochemical status and risk of overall and cause-specific mortality, a prospective cohort analysis was conducted using participants in the ATBC Study (1985–1993) previously selected for metabolomic sub-studies. The analysis included 4468 participants, among whom 3377 deaths occurred during an average of 19.1 years of follow-up. Serum erythritol was assayed using an untargeted, global, high-resolution, accurate-mass platform of ultra-high-performance liquid and gas chromatography. Cause-specific deaths were identified through Statistics Finland and defined by the International Classification of Diseases. After adjustment for potential confounders, serum erythritol was associated with increased risk of overall mortality (HR = 1.50 [95% CI = 1.17–1.92]). We found a positive association between serum erythritol and cardiovascular disease mortality risk (HR = 1.86 [95% CI = 1.18–2.94]), which was stronger for heart disease mortality than for stroke mortality risk (HR = 3.03 [95% CI = 1.00–9.17] and HR = 2.06 [95% CI = 0.72–5.90], respectively). Cancer mortality risk was also positively associated with erythritol (HR = 1.54 [95% CI = 1.09–2.19]). The serum erythritol–overall mortality risk association was stronger in men ≥ 55 years of age and those with diastolic blood pressure ≥ 88 mm Hg (*p* for interactions 0.045 and 0.01, respectively). Our study suggests that elevated serum erythritol is associated with increased risk of overall, cardiovascular disease, and cancer mortality. Additional studies clarifying the role of endogenous production and dietary/beverage intake of erythritol in human health and mortality are warranted.

## 1. Introduction

Erythritol ((2R,3S)-butan-1,2,3,4-tetrol) belongs to the family of sugar alcohols also known as polyols, which are formed through the hydrolyzation of aldehyde or ketone groups in various carbohydrates [1]. It is found naturally in some mushrooms, fruit (including grapes, watermelon, and pears), and fermented foods including wine, beer, and cheese, and has also been used as an artificial sweetener for over three decades [2]. Erythritol is excreted unchanged in the urine, and only a small percentage is metabolized to erythronate [3]. Along with aspartame and saccharin, erythritol is generally considered safe by regulatory agencies such as the U.S. Food and Drug Administration [4] and in the European Union [5], although there have been questions and studies regarding potential adverse health effects, including cancer and metabolic disorders [6,7,8,9].

Recently, some epidemiologic studies found artificial sweeteners to be associated with a risk of incident cardiovascular disease (CVD) independent of traditional CVD risk factors. A prospective interventional study showed that erythritol is clinically associated with increased cardiovascular event risks, noting that the sweetener enhances thrombosis potential in vitro and in animal models [10]. In targeted metabolomics analyses, serum erythritol was associated with a 3-year incidence of major adverse cardiovascular events, including fatal and nonfatal myocardial infarction or stroke [11]. The positive association between erythritol and the risk of major adverse cardiovascular event incidence was observed in both men and women, and was consistent across multiple population subgroups in both U.S. and European cohorts [11]. The results from the French-population-based cohort NutriNet-Sante suggested that higher total artificial sweetener use (i.e., aspartame, acesulfame potassium, and sucralose) is associated with increased risk of CVD [7], and the use of aspartame and acesulfame potassium with increased risk of overall cancer incidence [8]. Relatively few studies have been conducted on cancer risk compared to studies on heart disease risk, but it has been reported that there is a potential relationship between erythritol and liver cancer [12]. As studies of serum erythritol and long-term overall and cause-specific mortality have not been conducted, however, we endeavored to examine these associations in the present investigation.

## 2. Materials and Methods

### 2.1. Study Population

The Alpha-Tocopherol, Beta-Carotene Cancer Prevention (ATBC) Study consisted of male Finnish smokers aged 50 to 69 years who were assigned to receive either vitamin E (dl-α-tocopheryl-acetate, 50 mg/day), beta-carotene (20 mg/day), both vitamins, or a placebo for 5–8 years (a median of 6.1 years) [13]. At enrolment, participants completed questionnaires regarding behavioral and lifestyle information, including number of cigarettes smoked daily, years of smoking, history of diabetes (yes, no), and physical activity in leisure time (heavy, moderate, light). Height, weight, and systolic and diastolic blood pressure were measured. Total and high-density lipoprotein cholesterol concentrations were measured through enzymatic assays [14]. High-performance liquid chromatography was used to determine serum α-tocopherol, β-carotene, and retinol concentrations [15]. Participants in the ATBC Study previously selected for metabolomic sub-studies of cancer risk, vitamin supplementation, and other phenotypes were included in this analysis (*n* = 4468; comprising 2606 cancer cases and 1862 non-cases). These included three studies of vitamin supplementation [16,17,18]; four prostate cancer studies [19,20,21,22]; one study each of pancreatic cancer [23], glioma [24], and hair dye use [25]; and other participant data from unpublished studies.

The ATBC Study was approved by institutional review boards at both the U.S. National Cancer Institute and the Finnish National Public Health Institute, and participants provided written informed consent. All methods were performed in accordance with the relevant guidelines and regulations.

### 2.2. Serum Erythritol Measurement

In the ATBC, blood samples were collected from all participants who had been fasting up to 12 h at the time of their first baseline visit, which were stored at −70 °C. Baseline serum metabolite data that included erythritol were assayed at Metabolon, Inc. (Durham, NC, USA) using untargeted, global HD4 high-resolution, accurate-mass platform of ultra-high-performance liquid chromatography/tandem mass spectrometry (LC-MS/MS) and gas chromatography/tandem mass spectrometry (GC-MS/MS). Erythritol values were log-transformed and batch-normalized; methodological details of the metabolomic assays have been described [16,25,26]. In order to ensure accurate and consistent identification of true chemical entities and to exclude those that represented misassignments, background noise, and system artefacts, quality control (QC) and curation procedures were applied throughout the assay procedures. The relative standard deviation (RSD) of the internal standards added to each sample prior to injection into the mass spectrometers was calculated to estimate the instrument variability.

### 2.3. Mortality Endpoints

Deaths were identified through linkage with Statistics Finland. Cause-specific mortality was defined by three International Classification of Diseases for Oncology code (ICD) versions: cancer (ICD8/9: 140–208; ICD10: C00–C97), cardiovascular disease (ICD-8, 390–458; ICD-9, 390–459; and ICD-10, I00–I99), stroke (ICD8/9: 430–438; ICD10: I60–I69), and heart disease (ICD-8 and ICD-9, 390–398, 401–404, 410–429, and 440–448; ICD-10: I00–I13, I20–I51, and I70–I78).

### 2.4. Statistical Analysis

We conducted multivariable Cox proportional hazards regression models to investigate the associations between log-transformed serum erythritol and risk of overall and cause-specific mortality. The proportional hazards assumption was tested and met using a erythritol–follow-up time cross-product term. Follow-up time for each participant was calculated from date of baseline randomization to date of death or mortality censoring date (31 December 2013), whichever occurred first. Age, body mass index, systolic blood pressure, diastolic blood pressure, number of cigarettes smoked daily, years of smoking, serum total and high-density lipoprotein (HDL) cholesterol, diabetes mellitus (yes, no), and physical activity (heavy, moderate, light) were adjusted. Subgroup analyses were conducted for age, body mass index (BMI), number of cigarettes smoked daily, years of smoking, systolic blood pressure, diastolic blood pressure, and serum total cholesterol. To check the robustness of the findings, we additionally adjusted for when the metabolite assays were conducted and for variables that differed significantly between deceased and living participants. Kaplan–Meier survival plots of overall and cause-specific mortality according to erythritol quartiles were generated. The log-rank test was used to test the null hypothesis of no difference in survival between the erythritol categories.

All the analyses were performed using SAS statistical software version 9.4 (SAS Institute Inc., Cary, NC, USA). All reported *p* values are two-sided with 0.05 as the statistical significance threshold.

## 3. Results

The analysis included 4468 participants, among whom 3377 deaths occurred during an average follow-up of 19.1 years. Compared to those who died from any cause, survivors were younger, taller, and weighed more; had lower systolic blood pressure, fewer cigarettes smoked per day and years of smoking, higher serum retinol, serum α-tocopherol, and β-carotene; and were less likely to have a history of diabetes or being more physically active (Table 1).

After adjustment for several important potential confounders, higher serum erythritol was associated with increased risk of overall mortality (HR = 1.50 (95% CI = 1.17–1.92; *p* = 0.001); Table 2). The examination of cause-specific mortality revealed a positive association for CVD mortality risk (HR = 1.86 (95% CI = 1.18–2.94; *p* = 0.008) for erythritol as a continuous variable; HR 1.21 (95% CI = 1.02–1.45; *p* = 0.02) for fourth versus first quartile). The association was stronger for heart disease than for stroke: continuous HR = 3.03 (*p* = 0.05) and HR 2.06 (*p* = 0.18), respectively. Cancer mortality risk was also positively associated with serum erythritol (continuous HR = 1.54 (95% CI = 1.09–2.19; *p* = 0.02)). The analysis of erythritol quartiles showed Q4 versus Q1 HRs of 1.33, 1.20 and 1.17 for heart disease, stroke, and cancer mortality risk, respectively. Excluding the first five years of follow-up yielded similar findings (e.g., heart disease mortality HR = 2.04 and cancer mortality HR = 1.42). Additional adjustment for when erythritol and other metabolites were assayed and for variables differing significantly by vital status in Table 1 yielded similar or somewhat stronger HRs. Furthermore, when the models were additionally adjusted for energy intake (continuous, kcal/day) and sugar intake (sum of mono- and disaccharides, continuous, g/day), the results were consistent with our primary findings. Since erythritol is known to be a pentose phosphate pathway (PPP) metabolite, we additionally analyzed the associations between other sugar molecules of the PPP and overall and cause-specific mortality. We observed elevated risk for some of the metabolites, including D-glucose, D-ribose, and D-xylulose (Supplementary Table S1).

Examining the serum erythritol–mortality risk association within risk factor subgroups, we found a significant effect modification by age (*p*-value for interaction = 0.003) and diastolic blood pressure (DBP) (P for interaction = 0.01), with stronger associations in men ≥ 55 years old (continuous HR = 2.27 versus 0.93 for younger men) and those with DBP ≥ 88 mmHg (continuous HR = 2.07 versus 1.06 for men with lower DBP) (Table 3). Although there was no statically significant evidence of interactions with other risk factors, the positive risk association for overall mortality risk appeared more prominent among non-diabetics and participants with higher BMI, years of smoking, and systolic blood pressure. The positive associations for CVD mortality were also more prominent among participants with BMI ≥ 26.0 kg/m^2^ (continuous HR (95% CI) =2.31 (1.26–4.24) than among those with lower BMI (HR (95% CI) =1.44 (0.72–2.89). Regarding the association between serum erythritol and the risk of cancer mortality, borderline statistical significance was observed among participants with a higher BMI (continuous HR (95% CI) = 1.60 (0.98–2.62); *p*-value = 0.06) versus 1.49 (0.91–2.45) for lower BMI.

**Figure 1:**
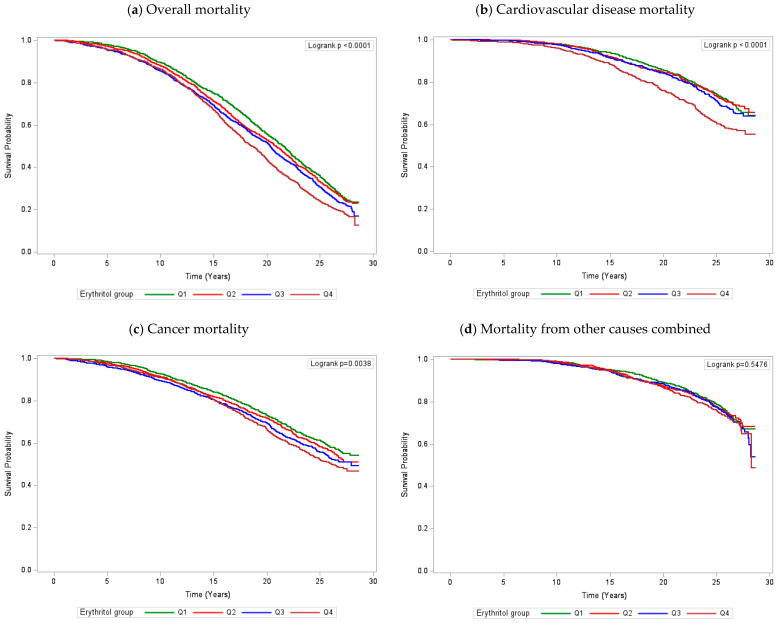
Kaplan–Meier plots of overall and cause-specific mortality according to serum erythritol quartiles.

Kaplan–Meier survival analysis demonstrated that participants in the highest quartile of serum erythritol experienced the lowest cumulative survival overall, except for non-CVD, non-cancer deaths (Figure 1). The log-rank tests indicated significant differences between four erythritol category survival curves for overall, CVD, and cancer mortality (*p* < 0.05).

## 4. Discussion

In the present study, higher serum erythritol was associated with increased risk of overall mortality. The association was strongest for heart disease mortality, followed by stroke and cancer mortality, and appeared evident primarily in older men and those with higher diastolic blood pressure.

A recent prospective analysis found that erythritol was associated with an increased 3-year incidence of major adverse cardiovascular events, particularly among participants aged 70 years and older [11]. However, in a long-term rat study with a daily erythritol dose up to 5.2 g/kg for 2 years, erythritol exposure did not affect kidney function, cancer incidence, or survival [27]. Moreover, a small pilot study that examined the effects of erythritol on vascular function in 24 patients with type 2 diabetes mellitus showed that daily intake of 36 g/day for 4 weeks led to reduced arterial stiffness and improved endothelial function [28], although the small study population was limited to patients with type 2 diabetes mellitus. The findings of the few previous studies on artificial sweeteners and risk of CVD and cancer are consistent with our findings, but they were not focused on erythritol specifically. A recent meta-analysis of randomized controlled trials and observational studies revealed that artificial sweeteners were associated with adverse cardiometabolic phenotypes [29]. Studies examining erythritol were not included in this analysis, which focused on aspartame and saccharin, and the controlled trials were of relatively short durations (median follow-up of only 3–6 months) and unable to capture long-term health outcomes. A separate meta-analysis suggested that artificially sweetened beverage consumption was associated with overall and CVD (but not cancer) mortality [30], and other studies of aspartame, acesulfame potassium, and sucralose use suggested increased risk of CVD and cancer [7,8]. Our findings highlight the need for additional large, long-term cohort studies focused on CVD and other health risks related to erythritol.

Several lines of evidence support the associations between erythritol and CVD-related phenotypes, including cell culture, and in vivo and preclinical mechanistic studies. Erythritol increases the responsiveness of human platelets to agonists and activation in vitro, as well as thrombosis formation in mouse models [11]. The plasma levels of erythritol remained elevated for at least 2 days after intake of an erythritol-sweetened drink above the threshold associated with increase platelet reactivity and thrombotic potential [11]. It has been previously postulated that associations between elevated erythritol and increased disease risk may merely be the result of dysregulation in the PPP caused by impaired glycemia [31]. This is certainly possible, in that erythritol is both ingested from exogenous (i.e., dietary/artificial sweetener) sources and is endogenously synthesized through the PPP, specifically via erythrose-4-phosphate, an intermediate of the non-oxidative phase. Erythrose-4-phosphate is the precursor of erythrose, which is transformed into erythritol by NADPH-dependent reactions catalyzed by alcohol dehydrogenase 1 (ADH1) and sorbitol dehydrogenase (SORD) [32,33]. In the present study, serum erythritol levels reflect both sources, which cannot be distinguished; however, our measurement of other sugar molecules of the PPP did provide some evidence for associations with higher mortality, supporting a possible contribution by glycemic disorders (Supplementary Table S1).

It should be noted that in our study erythritol and other metabolites were measured based on serum collected after an overnight fast. In a prospective intervention study of erythritol ingestion in eight healthy volunteers, postprandial plasma erythritol levels were examined following consumption of an erythritol-sweetened drink (30 g) (comparable to a single can of commercially available artificially sweetened beverage or a pint of keto ice cream or other erythritol-containing foods). Plasma erythritol levels were low at baseline, but remained 1000-fold higher (millimolar levels) for hours after intake and remained substantially elevated for over 2 days in all participants examined [11]. In contrast, a fairly short erythritol elimination half-life rate (i.e., <1 h for doses up to 50 g erythritol) has also been reported [34]. Our findings should be interpreted with caution, and it is possible that increased erythritol levels during fasting may have served as a pre-existing indicator of endogenous metabolic production in individuals at risk of early death. Additional prospective research is needed to evaluate the impact of exogenous erythritol on mortality risk.

Our investigation is the first to explore the association between serum erythritol and overall and cause-specific mortality risk. Deaths were ascertained through established and validated national registries. Use of biochemical data from a reliable high-performance mass-spectrometry platform more accurately reflected internal erythritol exposure as compared with questionnaire estimates of dietary intake. At the same time, the homogenous nature of our male smoker population of European ancestry may limit the generalizability of our findings to other populations. We did not assess the dietary intake of erythritol, and it is unclear whether the observed associations can be attributed to erythritol consumption per se, given that serum erythritol reflects both exogenous and endogenous sources, the latter of which may involve impaired glycemia. Therefore, a causal relationship between erythritol and risk of death remains plausible but not certain based on our study. There were a limited number of deaths in several subgroups, including diabetics. Large-scale studies are needed to determine whether diabetes status affects the relationship between erythritol and mortality risk. In addition, erythritol was measured only once based on baseline serum. Metabolite measurements from several time points would be helpful for evaluating the variation in serum metabolite levels over time and establish a more stable estimate of chronic exposure, in the setting of large-scale, prospective epidemiological cohort studies using metabolomics approaches. In addition, since we used serum erythritol as assayed using untargeted, global HD4 high-resolution, accurate-mass platform of ultrahigh-performance LC+GC-MS/MS, the metabolite levels are quantitative only in relative terms and not absolute or quantitative. However, the erythritol measurements are reproducible with high precision. Even though we cannot provide quantitative concentrations per se, given that the purpose of this study was to examine the association between serum erythritol and the risk of mortality endpoints, the use of the relative erythritol measurement did not affect the validity of our findings, which do represent quantitative mortality risk estimates [35,36,37]. Lastly, unmeasured or residual confounding may have exited even though we adjusted for several key potential confounding factors in the analysis. Mendelian randomization analyses of erythritol-related genotypes and cause-specific mortality risk would be highly informative in this regard.

## 5. Conclusions

In conclusion, higher serum levels of erythritol were associated with increased risk of overall, cardiovascular disease, and cancer mortality in men. Our findings suggest the need for further large-scale prospective epidemiologic and possibly safety studies examining the long-term association between erythritol, especially exogenous erythritol, and the risk of health and mortality outcomes.

## Figures and Tables

**Table 1:** Selected baseline characteristics [mean ± standard deviation or percentage (%)] of study participants.

Characteristics	All Participants (N = 4468)	Deaths (N = 3377) ^1^	Non-Deaths (N = 1091)	*p*-Values
Serum erythritol ^2^	1.02 ± 0.35	1.04 ± 0.36	0.98 ± 0.32	<0.0001
Age (years)	57.8 ± 5.1	58.8 ± 5.1	54.9 ± 3.8	<0.0001
Years of follow-up	19.1 ± 7.0	16.7 ± 6.3	26.8 ± 0.9	<0.0001
Height (cm) ^3^	173.7 ± 6.2	173.5 ± 6.1	174.3 ± 6.3	<0.0001
Weight (kg) ^3^	79.3 ± 12.6	78.9 ± 12.8	80.3 ± 11.9	0.0007
Body mass index (kg/m^2^) ^3^	26.2 ± 3.7	26.2 ± 3.7	26.4 ± 3.4	0.06
Systolic blood pressure (mmHg) ^3^	141.5 ± 18.7	142.6 ± 19.0	137.9 ± 17.3	<0.0001
Diastolic blood pressure (mmHg) ^3^	87.2 ± 10.5	87.4 ± 10.7	86.9 ± 10.0	0.16
Cigarettes smoked per day	19.6 ± 8.5	19.9 ± 8.5	18.6 ± 8.5	<0.0001
Years of smoking	35.9 ± 8.7	37.2 ± 8.6	32.0 ± 8.1	<0.0001
Serum biochemistry				
Retinol (µg/L)	591 ± 127	588 ± 127	601 ± 125	<0.0001
Total cholesterol (mmol/L)	6.2 ± 1.2	6.2 ± 1.2	6.2 ± 1.1	0.87
HDL cholesterol (mmol/L)	1.2 ± 0.3	1.2 ± 0.3	1.2 ± 0.3	0.50
α-Tocopherol (mg/L)	11.9 ± 3.3	11.9 ± 3.3	12.2 ± 3.0	0.005
β-Carotene (µg/L)	226 ± 201	216 ± 198	256 ± 206	<0.0001
Alcohol consumption (ethanol g/day)	15.7 ± 19.8	16.0 ± 20.5	14.6 ± 17.5	0.03
Trial α-tocopherol supplementation (%)	48.3	48.2	48.4	0.93
Trial β-carotene supplementation (%)	50.6	51.0	49.6	0.43
Diabetes mellitus (%)	3.2	3.7	1.5	0.0002
Light leisure time activity (%)	39.2	40.6	34.7	<0.0001

^1^ Death from all causes. ^2^ Relative levels obtained from untargeted, global HD4 high-resolution, accurate-mass platform of ultrahigh-performance liquid chromatography/tandem mass spectrometry (LC-MS/MS) and gas chromatography/tandem mass spectrometry (GC–MS/MS). ^3^ One participant was missing this variable.

**Table 2:** Associations between log-transformed serum erythritol and risk of overall and cause-specific mortality among 4468 men ^1,2^.

Mortality Outcome	# Events	Q1 HR	Q2 HR (95% CI)	Q3 HR (95% CI)	Q4 HR (95% CI)	P for Trend	HR (95% CI) (Continuous)	*p*-Value
All causes	3375	1.00	1.02 (0.93–1.13)	1.06 (0.97–1.17)	1.15 (1.04–1.27)	0.003	1.50 (1.17–1.92)	0.001
Cardiovascular disease	983	1.00	0.93 (0.77–1.12)	0.97 (0.81–1.17)	1.21 (1.02–1.45)	0.02	1.86 (1.18–2.94)	0.008
Heart disease	168	1.00	0.99 (0.62–1.58)	1.05 (0.66–1.66)	1.33 (0.86–2.06)	0.17	3.03 (1.00–9.17)	0.05
Stroke	189	1.00	0.69 (0.45–1.08)	1.02 (0.68–1.52)	1.20 (0.81–1.79)	0.15	2.06 (0.72–5.90)	0.18
Cancer	1657	1.00	1.08 (0.94–1.24)	1.14 (0.99–1.31)	1.17 (1.02–1.35)	0.02	1.54 (1.09–2.19)	0.02
Other causes combined	735	1.00	1.03 (0.84–1.27)	1.05 (0.86–1.29)	1.02 (0.83–1.26)	0.80	1.07 (0.63–1.81)	0.80

Abbreviations: CI: confidence interval; HR: hazard ratio; Q: quartile. ^1^ Two participants missing data for body mass index, systolic blood pressure, or diastolic blood pressure were excluded. ^2^ Multivariable-adjusted models included age, body mass index, systolic blood pressure, diastolic blood pressure, number of cigarettes smoked daily, years of smoking, serum total and high-density lipoprotein cholesterol, diabetes mellitus (yes, no), and physical activity (heavy, moderate, low).

**Table 3:** Risk of overall mortality associated with serum erythritol within subgroups of participant baseline characteristics.

Subgroups ^1^	No. of Deaths	HR (95% CI) for Q4 versus Q1 ^2^	HR (95% CI) (Continuous) ^2,3^	P for Interaction ^2,3^
Age (years)				0.003
<55	803	0.95 (0.77–1.16)	0.93 (0.57–1.52)	
≥55	2572	1.36 (1.21–1.52)	2.27 (1.72–3.00)	
BMI (kg/m^2^)				0.08
<26.0	1710	1.02 (0.89–1.17)	1.21 (0.86–1.72)	
≥26.0	1666	1.30 (1.13–1.50)	1.85 (1.31–2.60)	
Number of cigarettes smoked daily				0.66
<20	1335	1.12 (0.96–1.30)	1.52 (1.02–2.26)	
≥20	2040	1.17 (1.03–1.32)	1.50 (1.10–2.04)	
Years of smoking				0.25
<37	1413	1.05 (0.91–1.22)	1.23 (0.85–1.80)	
≥37	1962	1.23 (1.08–1.41)	1.69 (1.22–2.34)	
Systolic blood pressure (mmHg)				0.35
<140	1525	1.14 (0.99–1.32)	1.35 (0.93–1.96)	
≥140	1850	1.15 (1.01–1.32)	1.60 (1.15–2.22)	
Diastolic blood pressure (mmHg)				0.01
<88	1650	0.97 (0.84–1.12)	1.06 (0.74–1.52)	
≥88	1725	1.36 (1.19–1.56)	2.07 (1.48–2.90)	
Serum total cholesterol (mmol/L)				0.84
<6.17	1681	1.16 (1.01–1.33)	1.56 (1.10–2.20)	
≥6.17	1694	1.13 (0.99–1.30)	1.39 (0.98–1.97)	
Diabetes mellitus				0.60
No	3250	1.16 (1.05–1.28)	1.52 (1.19–1.96)	
Yes	125	0.95 (0.52–1.71)	0.98 (0.26–3.69)	

Abbreviations: CI: confidence interval; HR: hazard ratio; BMI: body mass index; Q4: fourth quartile; Q1: first quartile. ^1^ Subgroups are based on population median values unless otherwise noted. ^2^ Adjusted for age, body mass index, systolic blood pressure, diastolic blood pressure, number of cigarettes smoked daily, years of smoking, serum total and high-density lipoprotein cholesterol, diabetes mellitus (yes, no), and physical activity (heavy, moderate, low). ^3^ Log-transformed serum erythritol.

## Data Availability

The data used in this study are available from the corresponding author, Dr. Demetrius Albanes, upon reasonable request due to the requirements of the European Union GDPR research data privacy regulations.

## Supplementary Materials

The following supporting information can be downloaded at: https://www.mdpi.com/article/10.3390/nu16183099/s1, Supplementary Table S1: Associations between log-transformed serum metabolites of the pentose phosphate pathway and risk of overall and cause-specific mortality among 4468 men.
