# Supplementary material for: Serum Erythritol and Risk of Overall and Cause-Specific Mortality in a Cohort of Men
Source: Nutrients. 2024 Sep 14;16(18):3099. doi: 10.3390/nu16183099 (PMC11434845; doi:10.3390/nu16183099)
Supplement: Supplementary file 1 [file nutrients-16-03099-s001.zip › nutrients-3169390-supplementary.pdf]

**Supplementary Table S1.** Associations between log-transformed serum metabolites of the pentose phosphate pathway and risk of overall and cause-specific mortality among 4,468 men[1]

| | **Detection rate** | **All causes** | **Cardiovascular disease** | **Heart disease** | **Stroke** | **Cancer** |
|---|---|---|---|---|---|---|
| | | **HR (95% CI)** | **HR (95% CI)** | **HR (95% CI)** | **HR (95% CI)** | **HR (95% CI)** |
| D-Fructose | 100% | 0.89 (0.72-1.10) | 1.13 (0.75-1.69) | 2.00 (0.76-5.22) | 0.75 (0.30-1.90) | 0.75 (0.55-1.02) |
| D-Glucose | 100% | 1.12 (0.69-1.80) | 2.71 (1.15-6.41) | 2.44 (0.32-18.41) | 0.66 (0.08-5.29) | 0.89 (0.45-1.75) |
| D-Ribose | 54.5% | 1.33 (1.16-1.52) | 1.07 (0.85-1.35) | 1.09 (0.63-1.89) | 1.69 (0.89-3.22) | 1.68 (1.36-2.07) |
| L-Ribulose | 30.8% | 1.10 (0.89-1.36) | 0.88 (0.60-1.30) | 2.29 (0.79-6.62) | 1.35 (0.55-3.28) | 1.11 (0.83-1.49) |
| D-Xylulose | 13.9% | 1.12 (0.86-1.45) | 1.68 (0.95-2.96) | 5.91 (1.14-30.72) | 2.05 (0.53-7.87) | 0.88 (0.63-1.21) |

Abbreviations: CI: confidence interval; HR: hazard ratio

[1] Multi-variable adjusted models included age, BMI, systolic blood pressure, diastolic blood pressure, number of cigarettes smoked daily, years of smoking, serum total and HDL cholesterol, diabetes mellitus (yes, no), and physical activity (heavy, moderate, low).
